# Lung Inflammation Signature in Post-COVID-19 TB Patients

**DOI:** 10.3390/ijms242216315

**Published:** 2023-11-14

**Authors:** Galina S. Shepelkova, Vladimir V. Evstifeev, Yuriy S. Berezovskiy, Ruslan V. Tarasov, Mamed A. Bagirov, Vladimir V. Yeremeev

**Affiliations:** 1 Central Tuberculosis Research Institute, Moscow 107564, Russia; vladimir-evstifeev@yandex.ru (V.V.E.); report-q@yandex.ru (Y.S.B.); etavnai@yandex.ru (R.V.T.); m.bagirov@ctri.ru (M.A.B.); 2 Moscow Regional Clinical Tuberculosis Center, Moscow 127055, Russia

**Keywords:** tuberculosis, COVID-19, inflammation, pathology, microRNA

## Abstract

Tuberculosis (TB) remains a leading cause of infectious disease mortality worldwide, despite the COVID-19 pandemic. The mechanisms by which SARS-CoV-2 affects tuberculosis progression have not yet been established. Here, we compared the level of inflammation in the wall of the tuberculoma and in the parenchymal lung tissue of 30 patients diagnosed with tuberculoma without a history of COVID-19 and 30 patients diagnosed with tuberculoma 3 months after COVID-19. We also characterized TB activity in these patients using a panel of TB-associated miRNAs. Histopathological changes were examined in the resection material, and the expression level of cytokine/chemokine genes was determined by qRT-PCR. In patients with a history of COVID-19, the histological data obtained suggested activation of tuberculosis. In the same group of patients, as opposed to those without a history of COVID-19, equally high levels of pro-inflammatory cytokines/chemokines were expressed both in the tuberculoma wall and in the periphery of the resected specimen. A full set of miRNAs (miR-191, miR-193a, miR-222, miR-223, miR-155, miR-26a, and miR-150) were downregulated in the sera of patients with TB and active COVID-19 co-infection compared to controls. Our observations indicate signs of tuberculosis activation resulting from COVID-19 infection.

## 1. Introduction

Despite favorable trends in recent years, tuberculosis (TB) remains a serious public health problem in Russia. Of particular concern is the spread of drug-resistant forms of TB (MDR/XDR). The prevalence of patients with MDR-TB with bacterial excretion at follow-up was shown to reach 18.1 per 100,000 in 2021, although it had been decreasing since 2017 [1]. According to the WHO (2021), Russia has transitioned out of the top 30 countries with a high TB burden but remains in the list of countries with a high TB burden, HIV infection burden, and level of MDR-TB [2].

The COVID-19 pandemic has seriously undermined the progress made in the fight against tuberculosis disease worldwide and in the Russian Federation. Tuberculosis is a destructive respiratory disease. According to the WHO, it is associated with increased susceptibility to COVID-19 infection and a worse prognosis in patients [3]. Several studies indicate that patients with co-infection of TB and COVID-19 experience a higher frequency of negative clinical outcomes [4,5,6,7]. A recent meta-analysis performed by Sheerin D. and co-authors, as well as more recent cohort studies, showed that the dysregulation pathway of immune responses shared by TB and COVID-19 results in a dual risk to COVID-19 severity and TB disease progression [8,9]. 

According to the Central Research Institute of Health and Human Resources (Moscow), the effectiveness of MDR-TB chemotherapy in 2016 was about 64% [10]. Supplementing standard chemotherapy with methods of surgical treatment of limited forms of pulmonary TB can increase the effectiveness of treatment up to 90–98% [11]. To ensure positive outcomes of surgical interventions, the main task is to establish a favorable preoperative background in patients during preoperative preparation, including intensive chemotherapy and pathogenetic methods of treatment [12]. The main phthisiosurgical interventions in our country are being performed for pulmonary tuberculoma (76.5%) [11].

Pulmonary tuberculoma (caseoma) is a voluminous, caseous-necrotic formation over 12 mm in diameter separated from adjacent lung tissue by a capsule [13,14]. A fibrous layer is formed around the caseous mass. This is surrounded by a compact layer of granules. Both the granulation and fibrous layers of the capsule are well defined in newly formed tuberculomas. Over time, the inner layer becomes thinner, discontinuous, and sometimes disappears. This layer is the morphological basis of this pathology and is specific to tuberculous inflammation [15].

Many authors have described diffuse alveolar damage associated with disseminated intravascular coagulation syndrome (DIC) of varying degrees of severity, as in other viral infections of the lungs (caused by, for example, SARS-nCoV or MERS-CoV) [16]. Bacterial infections to the best of our knowledge are not a common complication of COVID-19 but can develop in patients on invasive ventilation [17].

Infection with SARS-CoV-2, which causes a concomitant disease, exacerbates the severity and prognosis of diseases in surgical patients, requiring a correction in the treatment approach for these patients. Due to the destruction of the integrity of the vascular walls, an increase in the number of hematogenous disseminated forms of TB should be expected. This might be accompanied by an increase in the number of intraoperative complications associated with the pathology of the vascular wall [18].

As the pandemic has evolved, effective vaccines and new therapies have become available, and it has become increasingly important to gain a deep understanding of late complications in patients with a history of SARS-CoV-2 infection. Due to the high prevalence of respiratory failure and the need for mechanical ventilation in patients with severe disease, there is a growing concern about long-term pulmonary complications, primarily pulmonary fibrosis (PF) [19].

Recently, we published the results of our investigation on serum miRNAs as biomarkers of the TB course in surgical patients [20]. Based on these results, here, we used a panel of TB-associated miRNAs to characterize TB activity in surgical patients with pulmonary tuberculoma as well as in surgical patients with pulmonary tuberculoma and a history of COVID-19. Features of inflammatory reaction development in the lung tissues of patients with pulmonary tuberculoma and in the lung tissues of post-COVID-19 patients with pulmonary tuberculoma were assessed based on the data from histological examination and analysis of the expression of a number of pro-inflammatory cytokine/chemokine genes in biopsy specimens obtained during surgical intervention.

## 2. Results

### 2.1. Lung Histology of Post-COVID TB Patients

**Figure 1:**
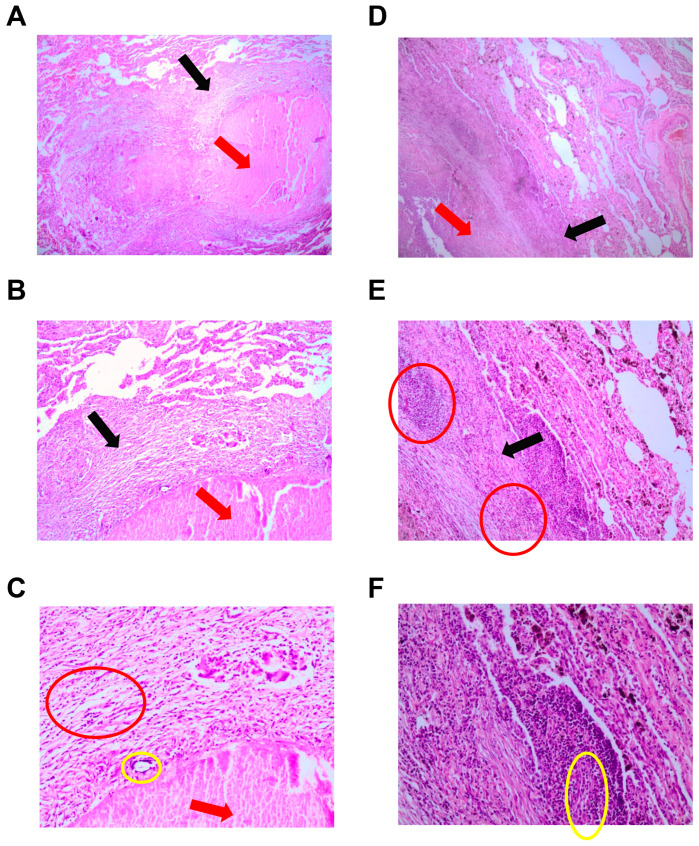
Comparison of the post-primary tuberculoma histopathology (**A**–**C**) and post-primary tuberculoma after 3 months post-COVID-19 (**D**–**F**). Human lung tissue. Hematoxylin and eosin staining. Magnification 90× (**A**,**D**); 200× (**B**,**E**); and 400× (**C**,**F**); red arrow—caseous necrosis; black arrow—fibrous capsule; red circle—lymphoid infiltrate; and yellow circle—blood vessel (**C**—with wall sclerosis; **F**—with inflammatory infiltrate in the wall).

**Figure 2:**
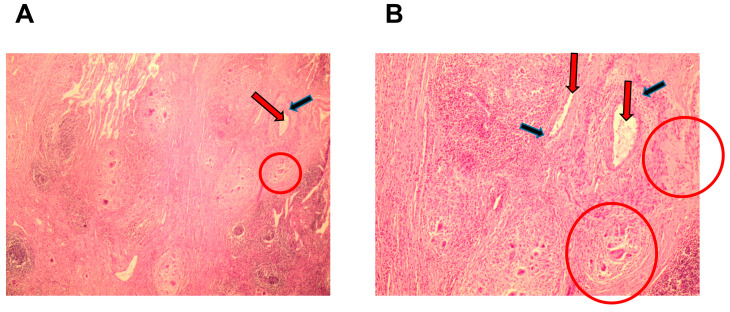
Penetration of granulomas into the vessel wall in active TB patients with tuberculoma 3 months post COVID-19. Human lung tissue. Hematoxylin and eosin staining. Magnification 90× (**A**) and 200× (**B**). Red arrow—blood vessel; black arrow—lymphoid infiltrate of the vessel wall; and red circle—granuloma.

**Figure 3:**
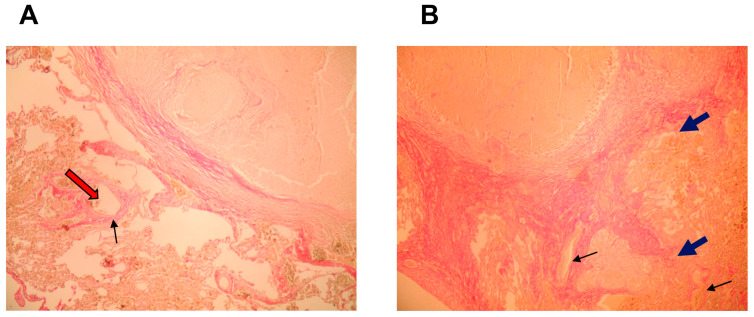
Comparison of fibrosis at the infection focus in lung tissue from a patient with active TB and tuberculoma (**A**) and from a patient with tuberculoma 3 months post COVID-19 (**B**). Human lung tissue. Van–Gieson staining. Magnification 90×. Red arrow—blood vessel; black arrow—blood vessel wall; and bold blue arrow—granulomatous inflammation.

**Figure 4:**
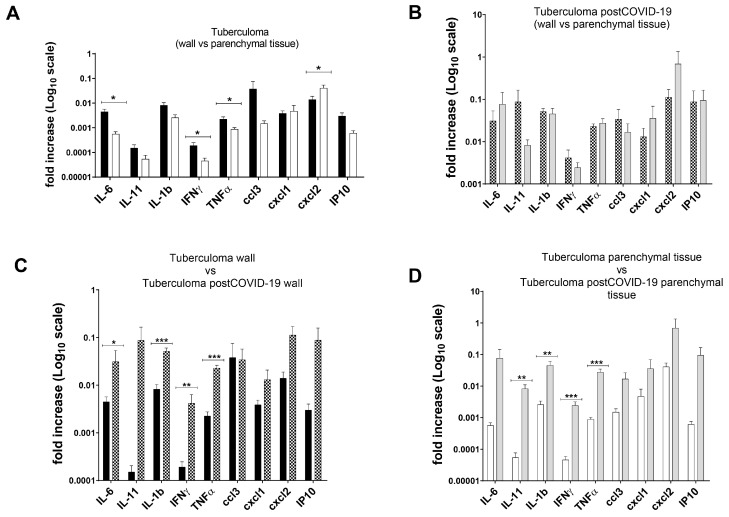
Co-infection with COVID-19 leads to increased inflammation in the lung tissue of patients with active TB even 3 months post viral infection. The cytokine/chemokine expression profile of the lung tissue of patients with tuberculoma (**A**) and tuberculoma 3 months post COVID-19 (**B**) is shown. (**C**) Comparison of cytokine/chemokine expression in the tuberculoma wall of patients without COVID-19 and after COVID-19. (**D**) Comparison of cytokine/chemokine gene expression in parenchymal lung tissue relatively distant from the site of infection on the scale of the operation. Gene expression levels were normalized to those of GAPDH. Black column—tuberculoma wall (patients with tuberculoma); white column—parenchymal tissue (patients with tuberculoma); plaid column—tuberculoma wall (patients with tuberculoma 3 months post COVID-19); and grey column—parenchymal tissue (patients with tuberculoma 3 months post COVID-19). Results of real-time PCR evaluation were quantified using the comparative threshold method and expressed as the mean fold increase ± SEM for 30 tuberculoma patients and 30 tuberculoma post-COVID-19 patients. * *p* < 0.05; ** *p* < 0.01; and *** *p* < 0.001.

Development peculiarities of inflammatory reactions in the lung tissues of patients with pulmonary tuberculoma and in the lung tissues of post-COVID patients with pulmonary tuberculoma were assessed based on the data from histological examination (Figure 1, Figure 2 and Figure 3) and analysis of the expression of a number of genes of pro-inflammatory cytokines/chemokines in biopsy specimens obtained during surgical intervention (Figure 4).

In tuberculoma patients, foci of caseous necrosis were combined in a mature connective tissue capsule (Figure 1A,B) with a noticeable lymphoid infiltrate and single epithelioid cell granulomas in the fibrosis phase (Figure 1C). Adjacent lung tissue showed mild focal lymphoid inflammation. Vessels displayed wall sclerosis (Figure 1C). In patients diagnosed with tuberculoma 3 months after suffering COVID-19, the focus of caseous necrosis was surrounded by a fibrous capsule with large areas of lymphoid inflammatory infiltrate (Figure 1D,E). In 100% of tuberculoma patients (30), extensive lymphoinflammatory infiltrates were observed in the fibrous capsule 3 months after COVID-19. In the adjacent lung tissue areas of patients with atelectasis, clusters of siderophages (large macrophages loaded with hemosiderin) were observed. These cells were brown in color; thus, there was no need for additional staining using specific dyes or antibodies. Such cells were found in all COVID-19 tuberculoma patients.

On histological sections of lung tissue from patients with active TB diagnosed with tuberculoma 3 months after suffering COVID-19, penetration of the granuloma into the vessel wall was observed against the background of the existing inflammatory lymphoid infiltrate of the wall (Figure 2A,B), which does not occur in the common TB course. There was “ingrowth” of the granuloma into the vessel wall in 24 patients with tuberculoma who had COVID-19 (80% of patients). In the normal course of TB, not complicated by SARS-CoV-2 infection, the vessels in/at the focus of infection were sclerosed, and there was no granulomatous reaction in the vessel walls. 

Specific Van–Gieson staining also revealed a number of histological differences between active TB patients without COVID-19 and active TB patients 3 months after COVID-19. Thus, Figure 3A (lung tissue of patients diagnosed with tuberculoma) shows a common TB lung image with a fibrous capsule surrounding the focus of dense caseous necrosis. Close to the focus of infection, there is a blood vessel with wall sclerosis. Patients with tuberculoma and a history of COVID-19 had a different histological lung tissue image. In Figure 3B, the destruction of mature connective tissue due to the increasing granulomatous inflammation is evident. Equal-sized fresh granulomas appear inside and outside the fibrous (connective tissue) capsule. Emerging foci of dissemination destroy the zone of perivascular fibrosis (around the blood vessels). All post-COVID19 TB patients (30 patients) had interstitial fibrosis and inflammation.

### 2.2. Cytokine/Chemokine Genes Are Differentially Expressed in Lung Tissues of TB and TB/Post-COVID Patients

The cytokine/chemokine gene expression profile in the lung tissue of patients with tuberculoma and tuberculoma 3 months post-COVID-19 is shown on Figure 4. Upregulation of the IL-6, IFNγ, and TNF-α genes along with downregulation of the cxcl2 gene were registered in the tuberculoma wall of TB patients when compared with gene expression in distant parenchymal tissues of the same patients (Figure 4A). In post-COVID-19 TB patients, no statistically significant differences between cytokine/chemokine gene expression profiles in the tuberculoma wall and in distant parenchymal tissues were observed (Figure 4B). When tuberculoma cell wall gene expression patterns were compared between TB patients post-COVID-19 and TB patients without COVID-19 (Figure 4C), significant prevalence of IL-1, IL-6, Il-11, IFNγ, and TNFα gene expression in the post-COVID-19 group was found. The same type of comparisons of parenchymal tissue expression patterns (Figure 4D) demonstrated upregulation of the same cytokine genes (IL-1, IL-6, Il-11, IFNγ, and TNFα) in post-COVID-19 parenchyma.

### 2.3. Serum miRNA Expression Pattern of TB Patients 3 Months Post-COVID-19 Differs from That of TB Patients without COVID-19 Infection

**Figure 5:**
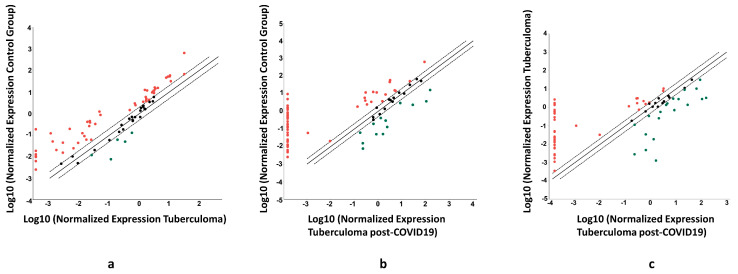
The difference in the expression of genes encoding mature miRNAs in the serum of active lung TB patients without COVID-19 and post-COVID-19 infection. Gene expression values as fold-changes in lung tuberculoma patients versus healthy controls (**a**), lung tuberculoma patients 3 months post-COVID-19 versus healthy controls (**b**), and lung tuberculoma patients versus lung tuberculoma patients 3 months post-COVID-19 (**c**). The median value for each gene from three independent replicates per group is presented in log10 scale. Red point—upregulated miRNAs; black point—unchanged miRNAs; green point—downregulated miRNAs; dotted line—the boundaries of the area in which the values are less than two times different from the comparison group; and solid straight line—values are identical to the comparison group.

The results of miRNA expression analysis with miScript miRNA PCR Arrays of the serum of TB patients are presented in Figure 5. The normalized expression patterns of 84 serum miRNAs in tuberculoma patients as compared with the control group (Figure 5a) differed from those of post-COVID-19 TB patients also compared with the control group (Figure 5b). Indeed, direct comparison of miRNA expression patterns between the tuberculoma group and the post-COVID-19 tuberculoma group showed that only a few miRNA genes were similarly expressed in these groups (Figure 5c).

**Figure 6:**
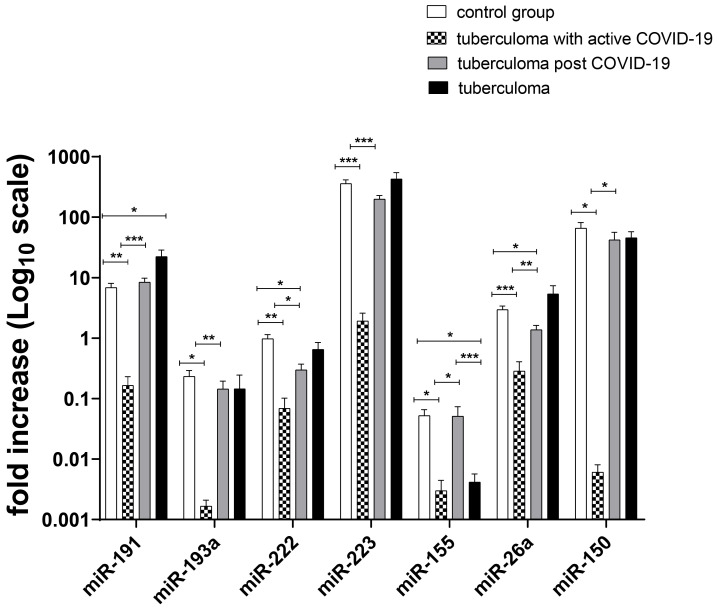
Expression of genes encoding mature miRNAs in the serum of the active lung TB group and the control group. Blood samples were collected from healthy controls (group 1—30 patients) and patients with tuberculoma (group 2—tuberculoma and active COVID-19 co-infection—20 patients; group 3—tuberculoma post-COVID-19, immediately after cessation of SARS-CoV-2 viral shedding in sputum—20 patients; group 4—tuberculoma without COVID-19—30 patients) and analyzed via qRT-PCR for miRNA expression. miR-103 was chosen as a reference for the data analysis of human samples. The mean ± SD values are shown. * *p* < 0.05; ** *p* < 0.01; and *** *p* < 0.001.

Further analysis of miRNA expression in the serum of TB patients by qRT-PCR using a set of markers that previously allowed us to reliably estimate the activity of specific inflammation in patients with tuberculoma [20] gave the following results, as presented in Figure 6. The levels of miRNA expression in the serum of healthy subjects, patients with tuberculosis without a history of COVID-19, and patients co-infected with tuberculosis and COVID-19 were evaluated. The co-infected group was tested twice: the first time was during active viral infection, and the second time was immediately after cessation of SARS-CoV2 shedding in sputum. The whole set of miRNAs (miR-191, miR-193a, miR-222, miR-223, miR-155, miR-26a, and miR-150) were downregulated in the sera of tuberculoma and active COVID-19 co-infection patients as compared with the control group (*p* values < 0.05; <0.01; and <0.001). At the point of sputum viral shedding cessation, most miRNA (miR-191, miR-193a, miR-222, miR-223, miR-26a, and miR-150) expression levels returned to that of tuberculoma patients without COVID-19 (Figure 6). The only exception was miRNA-155. miR-155 was downregulated in the tuberculoma group as compared with the healthy control group. Furthermore, at the point of sputum viral shedding cessation, no differences in miR-155 expression were registered between the post-COVID-19 group and healthy control group (Figure 6).

## 3. Discussion

The objectives of our study were to identify complications in patients diagnosed with TB who had COVID-19. Typical histological images of lung tissue in patients diagnosed with COVID-19 (postmortem) are detailed in our other article [21].

One of the typical histological manifestations of COVID-19 is the presence of hemorrhages of varying sizes and durations associated with viral invasion of the vascular wall. These hemorrhages occur both in the area of infection and in adjacent tissues [21]. Hemosiderophages are cells that can be a marker of viral infection for a long time and are responsible for clearing such bleeding. Such cells were found in all post-COVID-19 tuberculoma patients. In 100% of patients with tuberculoma, extensive areas of lympho-inflammatory infiltrate were observed in the fibrous capsule 3 months after COVID-19. In 24 patients (80% of patients) with tuberculoma who had COVID-19, there was “ingrowth” of the granuloma into the vessel wall. Interstitial fibrosis and inflammation were present in all post-COVID-19 TB patients.

SARS-CoV-2 infection is associated with the so-called “cytokine storm” [22]. We observed upregulation of the pro-inflammatory cytokine IL-6, IFNγ, and TNF-α genes and downregulation of the cxcl2 gene (a potent neutrophil chemoattractant) in the tuberculoma wall of TB patients compared with gene expression in distant parenchymal tissues of the same patients. This may reflect localization of the inflammatory process to the tuberculoma itself without involving surrounding tissues. On the contrary, there were no differences in expression levels of pro-inflammatory cytokine/chemokine genes between the tuberculoma wall and surrounding lung parenchymal tissues in post-COVID TB patients. This suggests a broader local inflammatory process compared to tissues from non-COVID TB patients. Direct comparison of pro-inflammatory cytokine gene expression patterns in the lung tissues of TB patients with those of post-COVID TB patients revealed upregulation of IL-1, IL-6, Il-11, IFNγ, and TNFα in both the tuberculoma wall and the lung parenchyma. Thus, the lung tissues of post-COVID TB patients were inflamed and this inflammation spread to adjacent tuberculoma tissues even 3 months after SARS-CoV-2 secretion had ceased (Figure 4).

In search of serum biomarkers of lung inflammation in TB tuberculoma patients with or without SARS-CoV-2 co-infection, we analyzed the dynamics of miR expression in patient blood samples. Inhibition of the relative expression of all microRNAs used in our study (miR-191, miR-193a, miR-222, miR-223, miR-155, miR-26a, and miR-150) at the peak of the COVID-19 disease reflects an imbalance in the host’s regulatory reactions during this period. As we demonstrated in Shepelkova et al. [20], this set of seven serum miRs could also be used during the course of preoperative chemotherapy to control TB lung destruction. Each of these miRs was evaluated for its role in TB infection. By regulating PMN migration and NF-kB activity, miR-223-3p can control excessive inflammation in TB [23]. MiR-26a-5p is able to attenuate the host immune response induced by TB infection and the IFNγ-dependent activation of macrophages [24]. MiR-26a and miR-191 have been shown to be differentially expressed in TB by Xuejiao Hu and co-authors [25]. It has been shown that hsa-miR-193a-3p is significantly upregulated in vitro in THP-1 cells infected with MTB [26]. MiR-222∗ is strongly down-modulated in human monocyte-derived macrophages infected with either virulent or avirulent mycobacteria [27]. Yung-Che Chen and co-authors have shown that gene expression levels of miR-150-5p are decreased in patients with active TB in comparison to either latently infected subjects or healthy controls [28].

Additional studies will be required to understand the patterns of miR-155 expression in the serum of patients with pulmonary TB and COVID-19. Several studies have implicated miR-155 in various viral infections and immune cell regulation [29,30]. Riham Abdel-Hamid Haroun and co-authors demonstrated an increased expression level of serum miR-155 in COVID patients as compared to healthy donors [31]. They also demonstrated a close correlation of the miR-155 expression level with clinicopathological characteristics of COVID-19 [31].

Thus, our studies showed that even three months after viral shedding had ceased, signs of inflammation associated with SARS-CoV2 infection persisted in the lungs of tuberculoma patients. This situation requires the surgeon to pay increased attention to the possibility of intraoperative bleeding and should be considered when deciding on the volume of lung tissue removed. Taken together, our data demonstrate an altered host ability to respond to and control MTB and/or SARS-CoV-2 upon co-infection, prompting further exploration of the immunological pathways underlying the immunopathogenesis of this combined disease.

## 4. Materials and Methods

### 4.1. Patients and Human Biological Material

The analysis included 80 patients with active pulmonary TB (tuberculoma) who were treated at the Central Tuberculosis Research Institute (Moscow, Russia) from October 2020 to March 2022. The criteria for enrolment were clinical and radiological findings (CT scan data) indicating active pulmonary TB (tuberculoma) including acid-fast bacilli (AFB) growth and/or GeneXpert^®^ positive results. Patients were divided into three groups:

- Thirty patients aged 18–65 years (12 women and 18 men)—tuberculoma without evidence of any clinical COVID-19 symptoms (with negative sputum SARS-CoV-2 PCR test) or history of COVID-19 or any other infections in the past three months;
- Thirty patients 20–65 years (17 women and 13 men)—tuberculoma 3 months post-COVID-19;
- Twenty patients 18–65 years (12 women and 18 men)—tuberculoma and active COVID-19 co-infection (including clinical COVID-19 symptoms and positive sputum SARS-CoV-2 PCR test).

Thirty age- and sex-matched patients with a negative history of TB and COVID-19 were recruited as a healthy control group. Individuals without evidence of any clinical TB and COVID-19 symptoms or history of TB and COVID-19 or any other infections in the past three months were included in the control group. All patients in the control group were tested for prior exposure to TB using skin immunological tests (Diaskintest^®^, GENERIUM, Moscow, Russia) [32]; the results were negative. All subjects in the control group had a negative serum SARS-CoV-2 IgG test. Individuals who were on any type of medication were excluded from the study.

Informed consent was obtained from all subjects involved in the study. This study was approved by the Institutional Ethics committee (IEC) of the Central Tuberculosis Research Institute (Moscow, Russia). All procedures involved and the complete design of the study were in accordance with the Declaration of Helsinki. 

### 4.2. Sample Collection and Processing

Two mL of blood was collected from 30 tuberculoma patients without evidence of any clinical COVID-19 symptoms, 30 healthy controls, and 20 patients with tuberculoma and active COVID-19 co-infection. In the case of patients with tuberculoma and active COVID-19 co-infection, blood samples were collected twice: during an active phase of the viral infection and immediately after cessation of SARS-CoV-2 viral shedding in sputum. Blood samples were centrifuged at 1500× *g* for 15 min at 4 °C. Sera were isolated and stored at −80 °C until use.

Biopsy surgical material of lung tissue was obtained from patients with tuberculoma (without and 3 months post-COVID-19) who underwent surgical treatment for TB. The biopsy material consisted of the tuberculoma wall and “healthy” parenchymal tissue on the scale of the operation.

Part of the biopsy material (tuberculoma wall and parenchymal tissue) was fixed into special aqueous, nontoxic tissue RNA stabilization and storage reagent–RNAlater™ Stabilization Solution (ThermoFisher Scientific (Invitrogen), Waltham, MA, USA) and used for cytokine and chemokine qRT-PCR expression analysis. Another part of the biopsy material was fixed in 10% buffered formalin (Sigma-Aldrich, Saint Louis, MO, USA) and then used for histological techniques.

### 4.3. Total RNA Extraction

TRIzol^®^ Reagent (ThermoFisher Scientific (Ambion), Waltham, MA, USA) was used for isolation of lung tissue high-quality total RNA. In all, 100 mg of fixed lung tissue was homogenized in 1 mL of TRIzol^®^ Reagent solution with a handheld homogenizer (Miulab, Zhejiang, China). Subsequent procedures for RNA isolation were performed in accordance with the manufacturer’s recommendations.

Blood serum total RNA isolation was conducted according to the previously described protocol [20]. Briefly, for monitoring miRNA purification and amplification, miRNeasy Serum/Plasma Spike-in Control (*C. elegans* miR-39 miRNA mimic) (QI-AGEN Gmbh, Hilden, Germany) was added to each serum sample before RNA extraction. Total RNA extraction from blood serum samples was performed using TRIzol LS (ThermoFisher Scientific (Invitrogen), Waltham, MA, USA) according to the manufacturer’s instructions. Subsequently, these RNA samples were used for miScript miRNA PCR Arrays (QIAGEN Gmbh, Hilden, Germany) and real-time PCR (qRT-PCR).

### 4.4. miRNA PCR Array

Serum samples were screened using the following strategy. Thirty samples of purified RNA (for each group–tuberculoma patients, tuberculoma 3 months post-COVID-19 patients, and healthy donors) were selected. RNA was pooled into three pools for each patient/donor group using equal volumes of each RNA. Each pool contained RNA from 10 samples. The miScript II RT Kit (QIAGEN, GmbH, Hilden, Germany) was used for the RT reaction. qRT-PCR was performed using the miScript miRNA PCR Array (QIAGEN GmbH, Hilden, Germany). QIAGEN’s GeneGlobe dedicated software (https://geneglobe.qiagen.com/ (accessed 12 November 2023))was used for data analysis.

### 4.5. Quantitative Real-Time PCR

qRT-PCR with cDNA was performed using the CFX96 Real-Time System (Bio-Rad; Hercules, CA, USA), specific primers, TaqMan probes, and reagents from ThermoFisher Scientific (Applied Biosystems, Waltham, MA, USA) to determine mRNA levels for inflammation-related genes. GAPDH was used as the housekeeping gene.

cDNA was synthesized and qRT-PCR for miRNAs (miR-191, miR-193a, miR-222, miR-223, miR-320, miR-18, miR-150, miR-155, and miR-26a) was performed according to a previously described protocol [20] using the TaqMan^®^ Advanced miRNA cDNA Synthesis Kit (ThermoFisher Scientific (Applied Bio-systems), Waltham, MA, USA) and Taq-Man Advanced miRNA Assays (ThermoFisher Scientific (Applied Biosystems), Waltham, MA, USA). For data analysis of human samples, miR-103 was selected as the reference [33].

### 4.6. Histological Staining

Paraffin sections of lung tissue were stained with hematoxylin and eosin (H&E) for lung inflammation evaluation. To evaluate pulmonary fibrosis, Van—Gieson staining was used. All samples were examined under a microscope (Leica DM2000, Leica Microsystems GmbH, Wetzlar, Germany).

### 4.7. Statistical Analysis

GraphPad Prism version 8.0.1 was used as a statistical tool for data analysis. Multiple t-test with multiple comparison (Sidak-Bonferroni method) was used to compare the fold changes between groups; p-values less than or equal to 0.05 were considered significant in all analyses. Data were presented as the mean ± SEM (standard error of the mean).

## Data Availability

The data are unavailable due to privacy or ethical restrictions.
